# Biphasic regulation of myogenesis by ALDH2, aldosterone, and oxidative stress

**DOI:** 10.1007/s00424-026-03185-w

**Published:** 2026-06-09

**Authors:** José Emmanuel Martínez-Cortés, Maikel Valle-Clara, Damaso Fernández-Hernández, Guillermo Ávila

**Affiliations:** Departamento de Bioquímica, Cinvestav, AP 14–740, México City, 07000 México

**Keywords:** Myoblast, Myotube, C2C12 cells, Myogenesis, Reactive oxygen species

## Abstract

Aldehyde dehydrogenase 2 (ALDH2) is a mitochondrial enzyme that metabolizes 4-hydroxynonenal (4-HNE), a lipoperoxidation-derived aldehyde that promotes oxidative stress. ALDH2 is therefore considered part of the antioxidant defense. On the other hand, the hormone aldosterone enhances the production of reactive oxygen species (ROS) in many cell types. During skeletal muscle development (myogenesis), myoblasts fuse to form multinucleated cells, called myotubes. In the present study, we investigated the potential of ALDH2 to regulate ROS production in C2C12 cells and its effects on myotube formation during *early* (0–3 days) and *late* (4–9 days) stages. Western blot results indicate that these cells actually express ALDH2. Interestingly, incubating cells (1.5–24h) with the pro-oxidant compounds H_2_O_2_, 4-HNE, aldosterone, and daidzin (ALDH2 antagonist) enhanced ROS production. In contrast, two ALDH2 agonists (Alda-1 and AD-5591) prevented, with similar potency, the 4-HNE– and aldosterone–induced oxidative stress. We next demonstrated that incubating cells with H_2_O_2_ (0.5–5 µM) induces a biphasic regulation of myotube formation, with early inhibition and late stimulation. This H_2_O_2_-induced biphasic regulation was also reproduced by aldosterone, daidzin, and another ALDH2 antagonist (CVT-10216). Consistent with their effects on oxidative stress, the ALDH2 agonists Alda-1 and AD-5591 eliminated the regulation of myoblast fusion by H_2_O_2_ and aldosterone. Additional data suggest that aldosterone acts through the mineralocorticoid receptor, and both calpain and p38-MAPK are also implicated. These findings indicate that ALDH2 is essential for regulating oxidative stress in myogenic cells and is involved in a signaling pathway that governs myoblast fusion through biphasic and opposing mechanisms.

**Graphical abstract:**

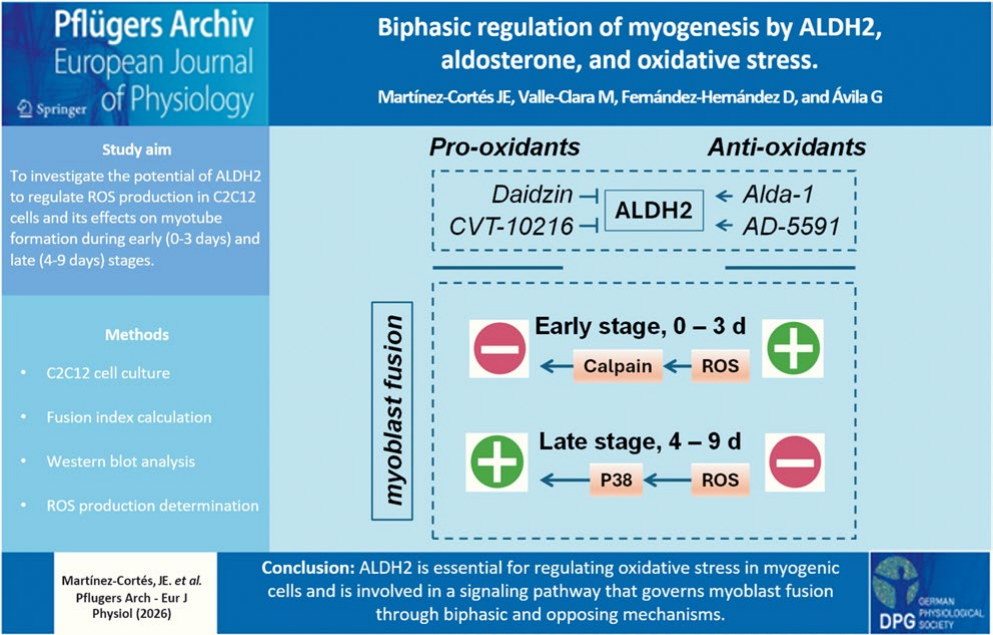

**Supplementary Information:**

The online version contains supplementary material available at 10.1007/s00424-026-03185-w.

## Introduction

Skeletal muscle development (myogenesis) involves the induction of coordinated changes in gene expression and a fusion of precursor cells (myoblasts) to form myotubes. That is, multinucleated cells that progressively express the muscle phenotype. Myoblast fusion is relevant in many physiological conditions, including muscular morphogenesis, hypertrophy, and regeneration. The fusion of myoblasts can be quantified in vitro by assessing the “fusion index”, consisting of the number of nuclei per myotube, divided by the total number of nuclei per field of observation. This indicator is critical for understanding the in vivo condition, because the size of the myofiber — and therefore the contractile force — depends on the number of nuclei within each fiber. In fact, muscle atrophy correlates with a lower number of nuclei per myofiber and, conversely, myonuclei accretion is required for muscle mass regeneration [3, 10, 20].

Earlier research suggests that oxidative stress exerts a two-phase effect on myotube formation: depending on the precise developmental phase, it can be either inhibitory (early stage) or stimulatory (late stage). The early, inhibitory effect has been observed in C2C12 cells exposed to aldosterone from day 0 to day 3 of differentiation (d0-d3). More precisely, aldosterone treatment results in high production levels of reactive oxygen species (ROS), as well as in low myoblast fusion index. However, the antioxidant N-acetylcysteine (NAC) blunts both effects [17].

The concept that ROS overproduction can stimulate myotube formation at slightly more advanced stages of differentiation, particularly in myotubes grown from day 4 (d4) to days 8–9 (d8-d9), is supported by the following evidence. First, Kosmidou et al. (2002) demonstrated that oxidative stress conditions drastically increases secretion levels of interleukin 6 (IL–6), in differentiated myotubes, but not in proliferating myoblasts [15]. Second, exposure of C2C12 myotubes to recombinant IL-6 results in elevated expression of the myogenic differentiation markers myogenin and α-actin [4]. Third, the exposure of C2C12 myotubes to recombinant IL-6 (from d4 to d8–9) results in a marked stimulation of myoblast fusion [29]. Thus, in the late stage of differentiation, oxidative stress activates an IL-6 autocrine mechanism that takes over to stimulate myotube formation.

The enzyme aldehyde dehydrogenase 2 (ALDH2) is part of the antioxidant defense because metabolizes reactive aldehydes, such as 4-hydroxynonenal (4-HNE), a compound derived from lipid peroxidation that modifies mitochondrial proteins and thereby induces ROS overproduction (for a recent review, see [1]). The compounds Daidzin and Alda-1 are amply recognized as ALDH2 regulators: The former is an isoflavone that binds to the catalytic site, acting as a competitive and reversible inhibitor, with an IC_50_ of approximately 80 nM. In contrast, Alda-1 acts allosterically and stimulates catalytic activity up to 100%. Interestingly, Alda-1 can reverse an ALDH2-inactivated state induced by the substrate 4-HNE, thereby optimizing enzyme activity even under oxidative conditions (reviewed in [8]).

Other “alternative” ALDH2 modulators have also been reported, such as the CVT-10216 (antagonist) and the AD-5591 (agonist). The latter is thought to act similarly to Alda-1. More precisely, through allosteric stimulation and binding to the same site as Alda-1, albeit with higher affinity. In keeping with this view, recent data indicate that AD-5591 exerts a greater effect on the enzyme than Alda-1, at the same concentration (100 µM [7]).

Interestingly, in myogenic cells, a putative ALDH2 has been identified through proteomic and mass spectrometry analysis [5, 18]. However, the identity of this macromolecule has yet to be corroborated, and its corresponding functional relevance should also be elucidated. Here, we found via western blotting that myogenic cells actually express ALDH2. Moreover, a systematic characterization of ALDH2 modulators’ effects indicates that this enzyme controls ROS production and, thereby, myotube formation at both the early and late stages of differentiation. Finally, in addition to identifying previously unrecognized functions of ALDH2, the present study provides evidence for a signaling cascade involving Aldosterone, ALDH2, 4-HNE, and ROS, which generates temporally opposite effects, thereby establishing a two-phase process in myogenesis.

## Methods

### C2C12 cell culture

The C2C12 cell line was obtained from the American Type Culture Collection (ATCC, Manassas, VA, USA; CRL-1772TM). A stock culture of proliferating cells was maintained in T-25 flasks, in the presence of “proliferation medium” consisting of: Dulbecco’s Modified Eagle’s Medium (DMEM, GibcoTM 12100–046) supplemented with 20% fetal bovine serum (FBS; Gibco® 16,000–044), 4 mM L-Glutamine (GibcoTM, 25,030), 100 U/mL penicillin, and 100 µg/mL streptomycin (GibcoTM 15140–122). The cells were kept incubated at 37 °C in a humid, saturated atmosphere with 5% CO_2_.

**Fig. 1:**
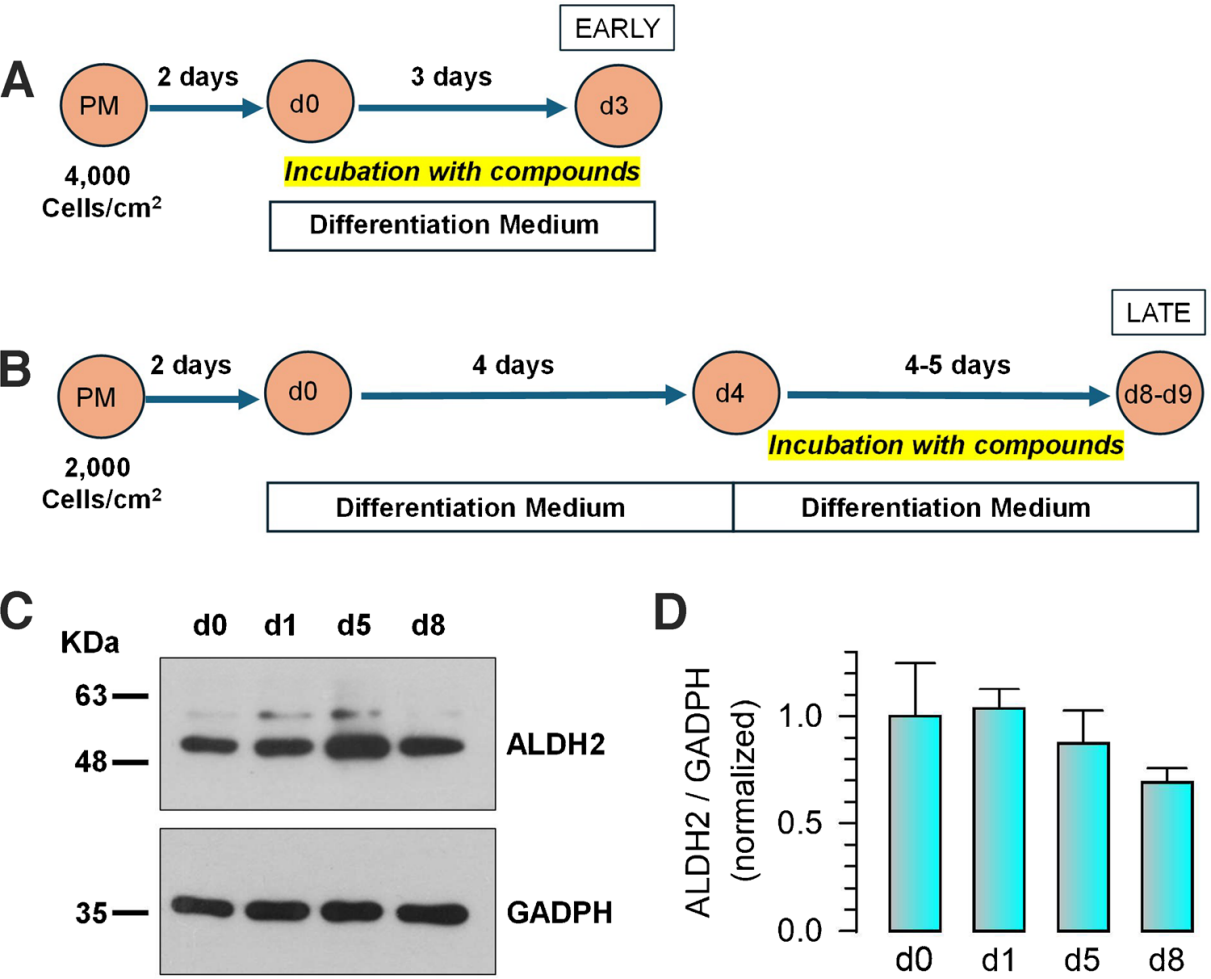
Cellular models for early and late skeletal muscle differentiation and ALDH2 detection by Western blot. **A, B**) Schemes depicting experimental details for two models of myogenesis. The main difference consisted in cells plated at high density (~ 4,000/cm^2^) for investigating the early stage (**A**, days 0–3), whereas those used for investigating the late stage (**B**) were seeded at low density (~ 2,000/cm.^2^, days 4–9). PM stands for proliferation medium. **C**) Example of Western blot showing the expression of ALDH2 and GADPH (loading control) in cells cultured as in **B** for distinct days of differentiation (d0–d8). **D**) Densitometric analysis obtained from immunoblots corresponding to the experimental conditions described in **C**. The absolute ALDH2/GADPH ratios were normalized to the mean observed at d0. Data represent the mean ± SEM from three independent replicates (*p* = 0.44, ANOVA)

The cells destined for differentiation were plated in P-35 dishes. Two days later, they were switched to “differentiation medium”, identical to the former, but with only 2% serum. This phase was termed differentiation day zero (d0), and then the experimental procedures diverged to generate the two working models, termed “early” (from 0 to 3 days of differentiation) and “late” (from 4 to 8–9 days of differentiation). This classification was based on methods reported earlier [17, 29] and is illustrated in Fig. 1A and B. More specifically:

A) *Early stage* (Fig. 1A). 40,000 cells were plated per dish (~ 4,000 cells/cm2) and, 2 days later, they were transferred to differentiation medium (d0) containing 2% horse serum, either without supplementation (control condition) or supplemented with compounds of interest. After three days (d3), the cells were observed with a microscope and photographed to quantify the fusion index as described below.
B) *Late stage* (Fig. 1B). For this model, 20,000 cells were plated per dish (~ 2,000 cells/cm2) and spent two days in proliferation medium, after which they were transferred to a differentiation medium containing 2% FBS (d0). On the day fourth (d4), the differentiation medium was renewed, and the experimental conditions were divided into either standard differentiation medium (Control) or this medium but containing the compounds under investigation. Cells were then kept in the incubator for an additional period of 4–5 days, so the total differentiation period was 8–9 days.

Aldosterone was used at a concentration of 100 nM, based on data from Lee and colleagues (2021), who showed that this hormone concentration exerts its maximal effects [17]. Concentrations used for other compounds are indicated in the corresponding figure legends.

### Fusion index calculation

To analyze myotube formation, cells were loaded with Calcein-AM (ION biosciences, 1071E) and Hoechst (Sigma, B2261). The loading procedure consisted of incubating cells with 5 µM calcein-AM for 10 min at room temperature. Subsequently, calcein-AM was removed, and cells were incubated with Hoechst (1 µg/ml in DMEM base) for 15 min at 37 °C. Finally, cells were rinsed with Ringer’s solution and observed with either a 40X (oil-immersion) or a 20X objective on an Olympus IX71 microscope equipped with a digital video camera (Nikon, DS-1000).

Calcein-loaded myotubes were observed with the aid of an epifluorescence cube consisting of the following filters: 470–490 nm (excitation), 505 nm (dichroic), and 515–550 nm (emission). One picture was taken and then a second one for the same field, but using any of the following excitation cubes, designed to reveal the Hoechst-stained nuclei: **A)** 335–345 nm (excitation) and 380 nm (dichroic)—blue signal; or **B)** 355–365 nm (excitation), 410 nm (dichroic), and 500–540 (emission)—blue-green signal.

Next, the matching images were assembled *offline*, and the fusion index was estimated by counting the number of nuclei within the calcein fluorescent myotubes and dividing it by the total number of nuclei (i.e., in myotubes plus myoblasts). The green calcein signal was digitally changed to red to improve the contrast between cytoplasm and nuclei.

### Western blot analysis

The possibility that myogenic cells express ALDH2 was examined in proliferating myoblasts (d0) and in cells cultured for 1, 5, and 8 days under control differentiation conditions (Fig. 1B). Briefly, cell extracts were obtained by exposing cells to 100 µl of RIPA lysis buffer containing 1% (w/w) Nonidet P-40, 0.5% (w/v) sodium deoxycholate, 0.1% sodium dodecylsulfate (SDS), 50 mM Tris–HCl (pH 8.0), and phosphatase/protease inhibitors. Subsequently, the total protein abundance was determined by the Bradford method, and 15 µg of protein extract was subjected to SDS-PAGE. The proteins were then transferred to a nitrocellulose membrane (Bio-Rad), which was next blocked with 4.5% skimmed milk in phosphate-buffered saline (PBS) and incubated overnight at 4 °C with agitation using an anti-ALDH2 polyclonal antibody (ABClonal, Cat. A11500; diluted 1:1000), or anti-GADPH (Sigma-Aldrich Cat. G8795; diluted 1:2500). The membrane was then incubated for 1 h at room temperature in presence of a secondary antibody (goat anti-rabbit IgG-HRP, Santa Cruz Biotechnology, sc-2004 diluted 1:25,000 or goat anti-mouse IgG-HRP, Santa Cruz sc-2005 diluted 1:25,000; respectively). Finaly, the antibody-tagged proteins were revealed by chemiluminescence, using the prime western blotting detection reagent (GE Healthcare, RPN2232).

### ROS production determination

The relative level of ROS production was assessed in cells cultured as in Fig. 1A (day 1) and B (day 5), and loaded with the ROS-sensitive fluorescence sensor CM-H2DCFDA (as described earlier [29]). In brief, cells were incubated with CM-H2DCFDA (6 µM) for 30 min at room temperature, rinsed, and incubated for 20 min at 37°C. Next, the cells were observed with a digital video camera (Nikon DS-1000) mounted on an Olympus IX71 microscope. Subsequently, the fluorescence of CM-H2DCFDA was photographed using the same excitation cube described above for calcein and the mean fluorescence value was estimated with *ImageJ*. Absolute fluorescence values were then averaged and divided by the mean value obtained from control cells (normalized fluorescence).

### Data analysis

Data are expressed as both bars with means ± SEM from the indicated number of experiments (n) and raw individual values corresponding to each bar (open circles). They were analyzed using the following software packages: *ImageJ* and *SigmaPlot*. Two groups were compared using Student’s t-tests, while for three or more conditions, one-way analysis of variance (ANOVA) was used, followed by post hoc Dunnett’s tests. All experiments were conducted at room temperature (22–24 °C). A recent article provides a more complete description of the methodology [29].

## Results

### ALDH2 immunodetection and its role in ROS production

Our main goal was to assess whether ALDH2 participates in ROS production and myogenesis, using compounds that regulate its enzymatic activity: Alda-1 and AD-5591 (activators) and daidzin and CVT-10216 (inhibitors). We initially decided to determine if myogenic cells indeed express ALDH2. Figure 1C shows a representative immunoblot, showing a protein of approximately 50 kDa, near the theoretically calculated molecular weight of ALDH2 (~ 56 kDa). This observation is in keeping with results published by the manufacturers of the primary antibody (ABclonal, Cat. A11500), showing that ALDH2 is present in several primary tissues and cell lines, as well as with results of our group in which another primary antibody was used, in adult rat cardiac cells [22]. Therefore, the immunoblot of Fig. 1C provides the first direct evidence that myogenic cells indeed express ALDH2, a notion previously inferred from proteomic studies [5, 18]. Moreover, we also compared the ALDH2 expression across distinct days of differentiation. Particularly, between cells differentiated by 0, 1, 5, and 8 days. As shown in Figs. 1C-D, the ALDH2 levels remain relatively stable throughout the entire period, with a slight tendency to decrease by d8, though no significant changes were detected (Fig. 1C and D).

**Fig. 2:**
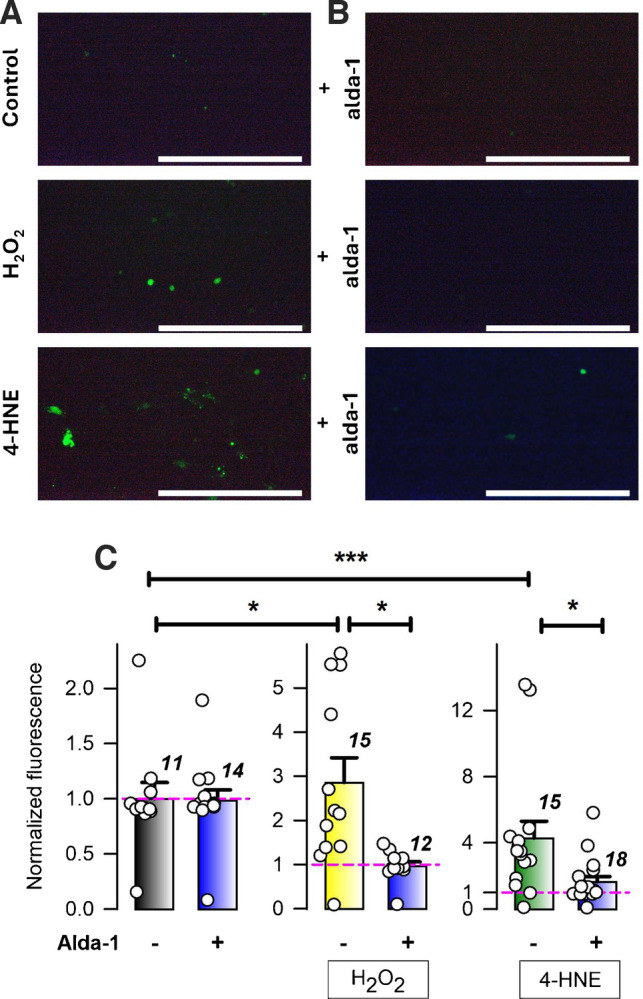
The ALDH2 agonist Alda-1 prevents acute pro-oxidant effects induced by H_2_O_2_ and 4-HNE. **A, B**) Fluorescence photographs obtained from cells cultured 1 day in standard differentiation medium (as in Fig. 1A) and then loaded with the ROS sensor CM-H2DCFDA. Approximately 90 min ahead of taking the pictures, cells were unexposed (***A***, *Control*) or exposed to 5 µM H_2_O_2_ or 10 µM 4-HNE, either in absence (**A**) or presence of 1 µM Alda-1 (**B**). Horizontal calibration bars represent 300 µm. **C**) Average values of ROS production that were estimated for the experimental conditions described in **A** and **B**. All data are shown normalized to the mean value from the control condition (dashed horizontal lines). Note that the three vertical axes are calibrated differently. ^*^p < 0.05, ^***^p < 0.001

The immunodetected protein (Figs. 1C and D) may regulate the redox state in C2C12 myotubes. To determine whether this is indeed the case, we studied the potential antioxidant effects of Alda-1 in both control cells and those exposed to oxidative challenges (5 µM H_2_O_2_ and 10 µM 4-HNE, for 90 min). The corresponding results are shown in Fig. 2. As expected, cells incubated with either H_2_O_2_ or 4-HNE showed higher ROS generation levels than controls (~ threefold and ~ fourfold, respectively). Remarkably, at a concentration of 1 µM, Alda-1 eliminated both effects without significantly altering basal ROS production (Fig. 2).

**Fig. 3:**
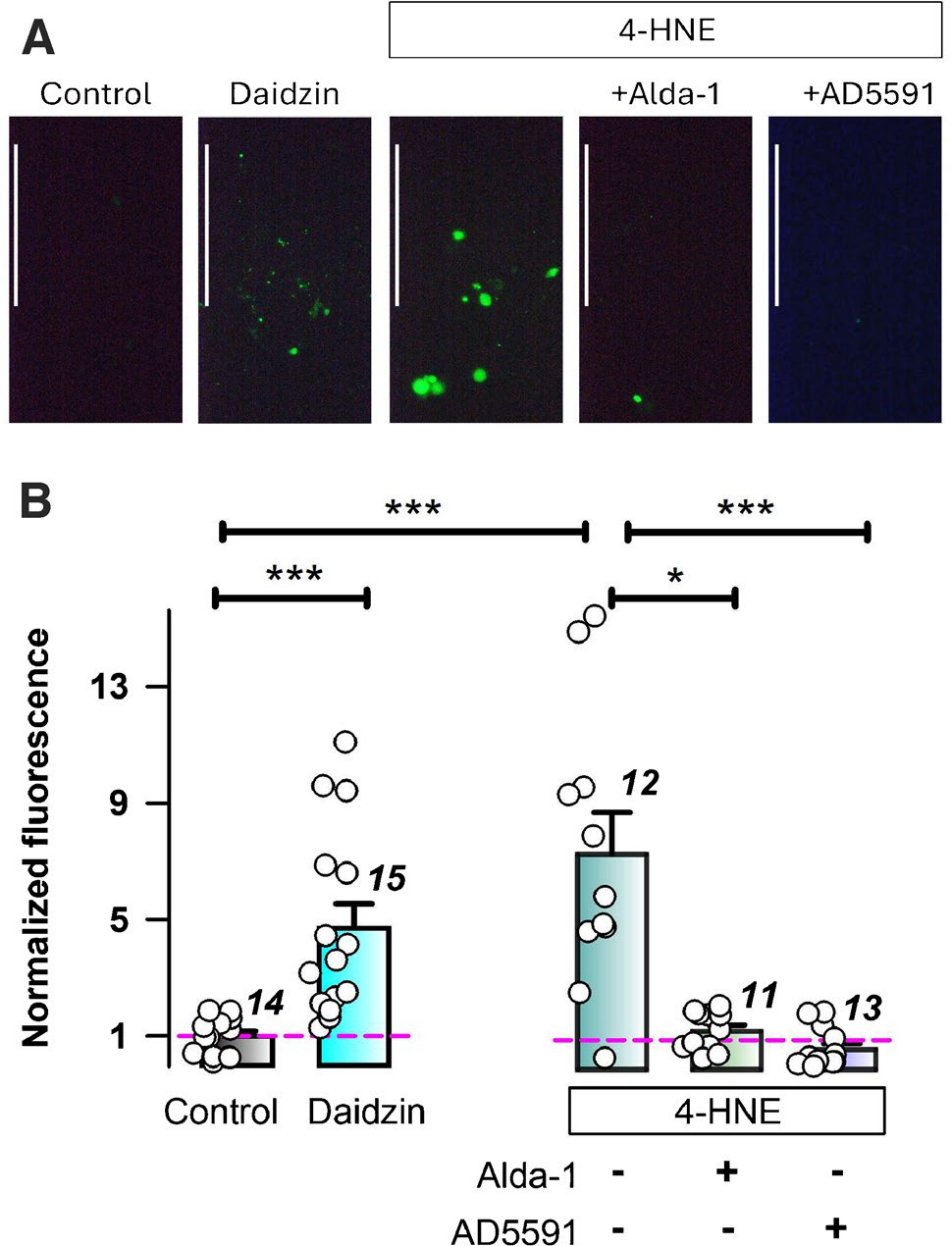
Long-term upregulation of ROS production by daidzin and 4-HNE and its prevention by Alda-1 and AD-5591. **A**) Representative epifluorescence images for the ROS sensor CM-H2DCFDA in cells cultured for 24 h in the absence (*Control*) or presence of 20 µM Daidzin or 10 µM 4-HNE (from d0 to d1). A subgroup of 4-HNE–treated cells was also exposed to 2 µM of either Alda-1 or AD-5591. Scale bars represent 300 µm. **B**) Average values of ROS production for experimental conditions described in **A**. Absolute values of ROS production were normalized to the mean observed in control cells (dashed lines). ^*^p < 0.05, ^***^p < 0.001

The following experiments were aimed at determining if the regulation of ROS production can persist beyond the acute effects of Fig. 2. In particular, in Fig. 3 we focused on cells exposed for 1 day (rather than 1.5 h) to pro-oxidant compounds (20 µM daidzin and 10 µM 4-HNE). In addition, subgroups of 4-HNE–treated cells were also incubated with 2 µM of either Alda-1 or AD-5591 (ALDH2 agonists). The experimental condition in which cells were exposed to only daidzin is critical because it could reveal a possible basal or constitutive activity of ALDH2. Remarkably, this was the case as the ALDH2 inhibitor daidzin considerably increased basal ROS production by nearly 400% (Fig. 3). Moreover, this long-term effect was mimicked by 4-HNE, indicating that the acute pro-oxidant effect of the latter (Fig. 2) prevailed even after 1 day of incubation (and indeed tended to grow, from fourfold to sevenfold; Fig. 2C and 3B). Perhaps more importantly, Fig. 3 shows that both ALDH2 agonists (Alda-1 and AD-5591) prevented this chronic oxidative stress (similar to what happened with the acute oxidative insult, Fig. 2).

**Fig. 4:**
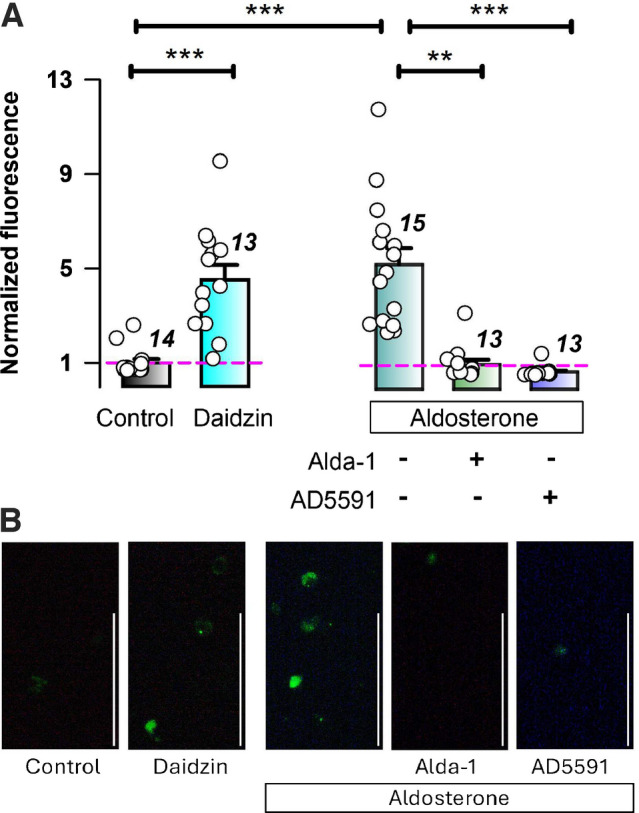
The ALDH2 agonists prevent chronic ROS production upregulation by aldosterone. **A**) Average values of ROS production estimated from cells cultured 1 d in control differentiation conditions and the presence of either 20 µM daidzin or 100 nM aldosterone. Subgroups of aldosterone-treated cells were also incubated with 2 µM of Alda-1 or AD-5591. Absolute ROS production values were normalized to the mean value observed in control conditions (dashed lines). ^**^p < 0.005, ^***^p < 0.001. **B**) Examples of images for the CM-H2DCFDA fluorescence that were used to estimate the ROS production levels of **A**. The calibration bar represents 150 µm

Our data show that H_2_O_2_, 4-HNE, and daidzin increase ROS production (Figs. 2 and 3). Likewise, previous work showed that the hormone aldosterone induces oxidative stress in C2C12 cells [17]. Thus, we next attempted to corroborate this aldosterone pro-oxidant effect, in cells treated 1 day, and using daidzin as the positive reference. As expected (see the data of Fig. 3), the ALDH2 antagonist raised again intracellular levels of ROS production by approximately 4.5-fold (Fig. 4, *daidzin*), and remarkably, this effect was mimicked by aldosterone, confirming that this steroid indeed promotes oxidative stress in myogenic cells (Fig. 4, *aldosterone*). More importantly, the compounds Alda-1 and AD-5591 readily prevented the aldosterone effect (Fig. 4, *Alda-1* and *AD-5591*), suggesting crosstalk between the aldosterone signaling and ALDH2.

**Fig. 5:**
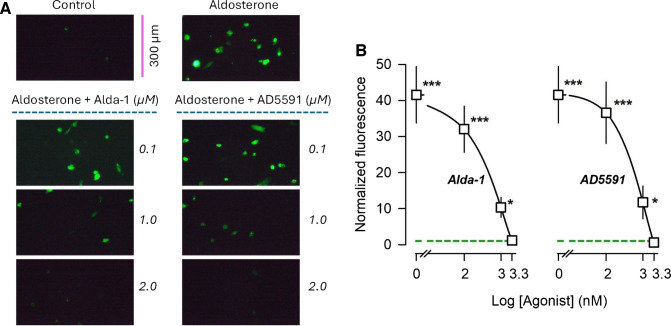
In the late model, the ALDH2 agonists Alda-1 and AD-5591 prevent oxidative stress induced by aldosterone with similar potency. **A**) Examples of ROS production levels for control cells and those treated from d4 to d5 with 100 nM aldosterone either alone or combined with distinct Alda-1 and AD-5591 concentrations (0.1 µM, 1.0 µM, and 2.0 µM). **B**) Average values of ROS production obtained from images as in **A**. Absolute fluorescence values were normalized to the mean obtained from controls, whose standard error (± 0.19) is illustrated as two dashed green lines around 1.0. ^*^p < 0.05, ^***^p < 0.001, compared to 0 µM (control). The n for each experimental condition ranges from 11 to 13

The above ROS measurements (Figs. 2, 3 and 4) were performed at d1 for the early model. We next assessed redox state on the first day of treatment for the late model. Specifically, we investigated if aldosterone increases ROS production in cells treated from d4 to d5 and whether ALDH2 agonists prevent this effect. Remarkably, aldosterone increased ROS production by about 40-fold compared with controls (Fig. 5; *Aldosterone*). More importantly, two micromolar of either Alda-1 or AD-5591 completely prevented this effect (Fig. 5). In this experimental series, we also included two lower agonist concentrations (0.1 µM and 1.0 µM) and found that both compounds had almost identical effects across all tested concentrations, indicating similar potency (estimated IC_50_ values of 259 nM and 388 nM for Alda-1 and AD-5591, respectively; Fig. 5).

### Roles of ALDH2 and ROS in the early phase of myogenesis

Thus far, our results indicate that ALDH2 activity is important for regulating ROS production in myogenic cells under both basal and pro-oxidant conditions. In the next experimental series, we set out to investigate the potential of ALDH2 and oxidative stress to regulate myogenesis.

**Fig. 6:**
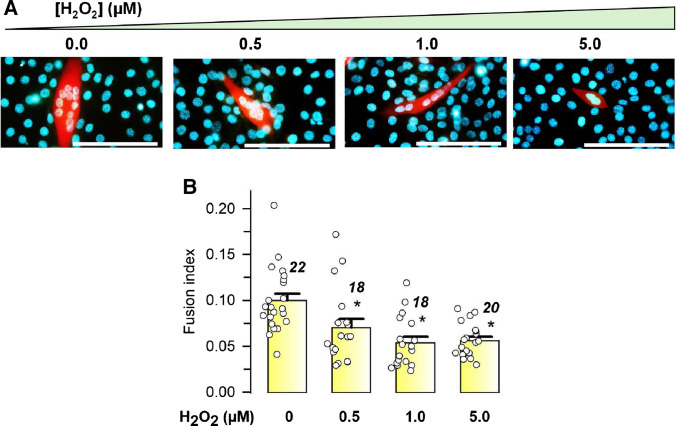
Peroxide hydrogen inhibits myoblast fusion in the early (3d) model of differentiation. **A**) Epifluorescence images that were assembled for Hoechst (blue) and calcein (red) to illustrate myonuclei distribution in cells cultured for three days in the presence of various concentrations of H_2_O_2_. Two images were taken for the same field of observation, but with different excitation cubes, designed to observe Hoechst (blue) and calcein (red) separately. Subsequently, each pair of photos was assembled to obtain a single image. Of note, in this and upcoming figures, the calcein signal was digitally converted from green to red to improve contrast and facilitate counting blue nuclei within red myotubes. Calibration bars account for 150 µm. **B**) Average values of fusion index obtained from images as those illustrated in **A**. ^*^p < 0.05, compared to 0 µM

**Fig. 7:**
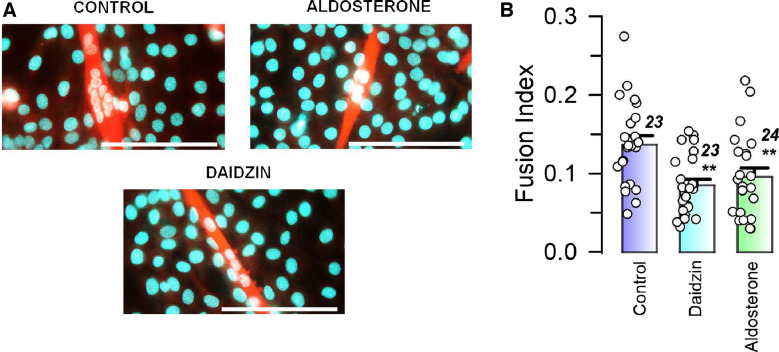
Aldosterone and daidzin mimic the early inhibitory effect of H_2_O_2_ on myotube formation. **A**) Representative images of myotubes that grew 3 d under control conditions and the presence of either aldosterone (100 nM) or daidzin (20 µM). Horizontal scale bars are equivalent to 150 µm. **B**) Average fusion index values estimated from images as shown in **A**. ^**^P < 0.01

For this, we initially investigated whether incubating cells with H_2_O_2_ could reproduce a previously reported inhibitory effect of aldosterone on myoblast fusion (originally attributed to ROS overproduction [17]). Specifically, we assessed myotube formation in cells exposed three days to different H_2_O_2_ concentrations. As can be seen in Fig. 6, hydrogen peroxide significantly reduced the fusion index at concentrations ranging from 0.5 µM to 5.0 µM (Fig. 6), corroborating the view that oxidant stress indeed inhibits muscle development at early stages of myogenesis [17]. Moreover, as shown in Fig. 7, the H_2_O_2_ inhibitory effect (Fig. 6) could be reproduced not only by aldosterone (in keeping with Lee et al., 2021) but also by daidzin (30–40% inhibition, Fig. 7). This inhibitory effect of daidzin, combined with its pro-oxidant action (Figs. 3 and 4), suggests that constitutive ALDH2 activity maintains low ROS production and thereby enables robust myoblast fusion during the early phase of myogenesis.

**Fig. 8:**
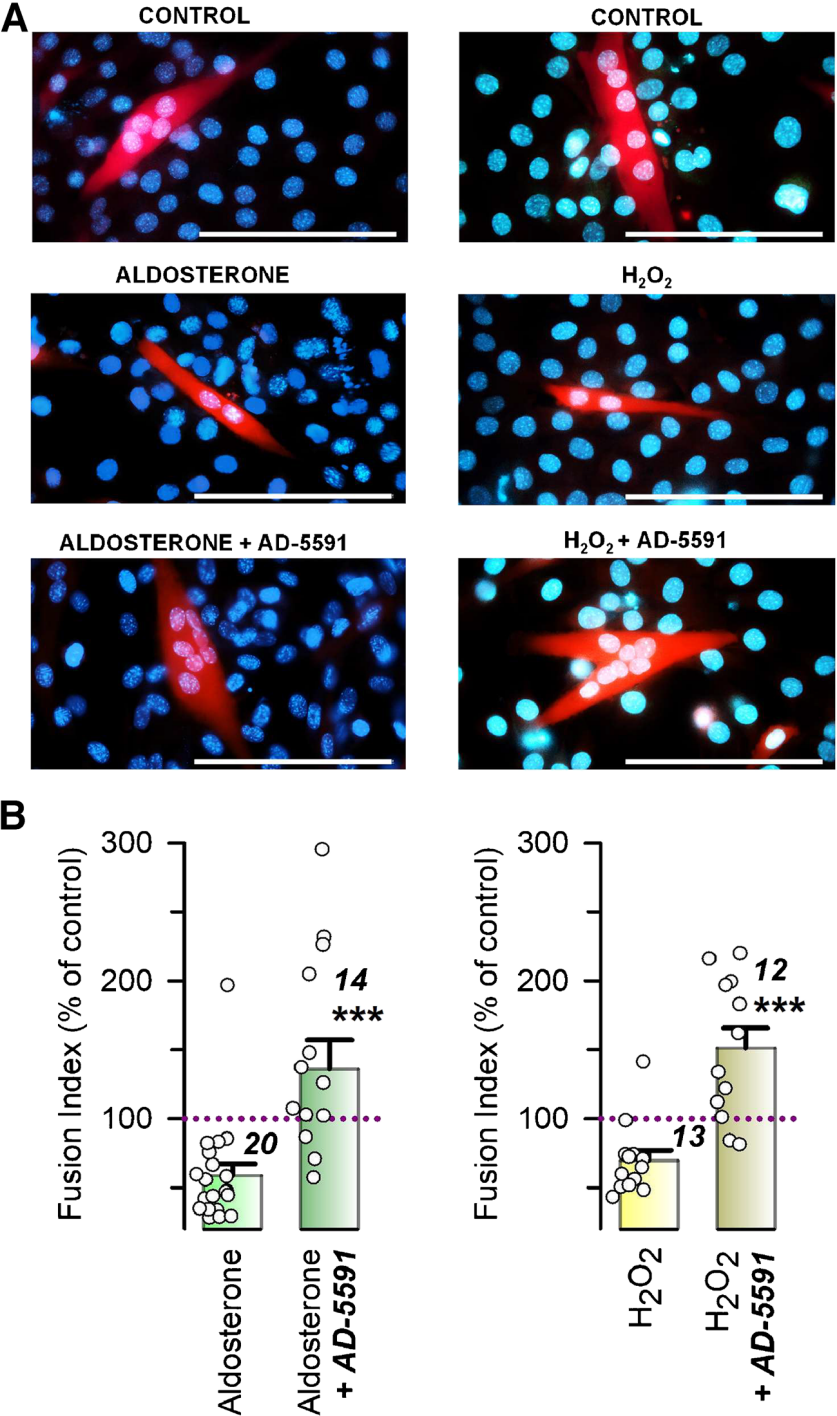
The ALDH2 agonist AD-5591 counteracts the early inhibition of myotube formation induced by aldosterone and H_2_O_2_. **A**) Epifluorescence images for Hoechst (*blue*) and calcein (*red*) obtained from cells cultured for three days under control conditions (*top*), the presence of aldosterone (100 nM, *middle-left*), H_2_O_2_ (1 µM, *middle-right*), or a combination of either of these compounds plus the ALDH2 agonist AD-5591 (2 µM, *low*). The scale bars represent 150 µm. **B**) Average fusion index obtained from myotubes cultured in the presence of aldosterone (*left*) and H_2_O_2_ (*right*), either alone or combined with AD-5591, as illustrated in **A**. Note that the data are presented as percentages relative to the absolute values for cells cultured under control conditions. The absolute values of fusion index obtained from the two Controls were, respectively: 0.11 ± 0.2 (*n* = 13, *left*) and 0.10 ± 0.1 (*n* = 22, *right*). ^***^p < 0.001

This view was corroborated using Alda-1. If the aldosterone inhibitory effect shown in Fig. 7 were due to oxidative stress (as inferred in [17], see also Fig. 4), then the antioxidant Alda-1 (Figs. 2, 3 and 4) should be able to enhance myoblast fusion in aldosterone-treated cells. In agreement with this prediction, cells co-incubated with aldosterone plus Alda-1 (1 µM) exhibited a 40% increase in fusion index, compared to those exposed only to aldosterone. Specifically, the fusion index obtained from cells exposed to only aldosterone (*n* = 17) and aldosterone plus Alda-1 (*n* = 21) was, respectively: 0.18 ± 0.02 and 0.25 ± 0.02 (*p* = 0.015). In addition to Alda-1, the idea that ALDH2 stimulation enhances myoblast fusion during the early stage of myogenesis was also challenged using AD-5591 (Fig. 8). It can be observed that like Alda-1, the ALDH2 agonist AD-5591 also upregulated myogenesis in aldosterone-treated cells, raising fusion index to values that were even higher than the corresponding controls (Fig. 8A and B, *left*). Additionally, AD-5591 drastically increased the fusion index in cells treated with H_2_O_2_, counteracting the limitation on myotube formation imposed by this molecule; and, again, enhancing myoblast fusion to levels even 50% larger than controls (Figs. 8A and B, *right*; see also Fig. 6). These data, combined with results shown in Figs. 2, 3, 4 and 5, indicate that the bidirectional regulation of myoblast fusion by aldosterone (inhibitory) and ALDH2 agonists (stimulatory) can be attributed to changes in ROS production. Summarizing, the above data indicate that in the early phase of differentiation, H_2_O_2_, daidzin, and aldosterone inhibit myoblast fusion by promoting oxidative stress, whereas ALDH2 agonists (Alda-1 and AD-5591) counteract these effects.

**Fig. 9:**
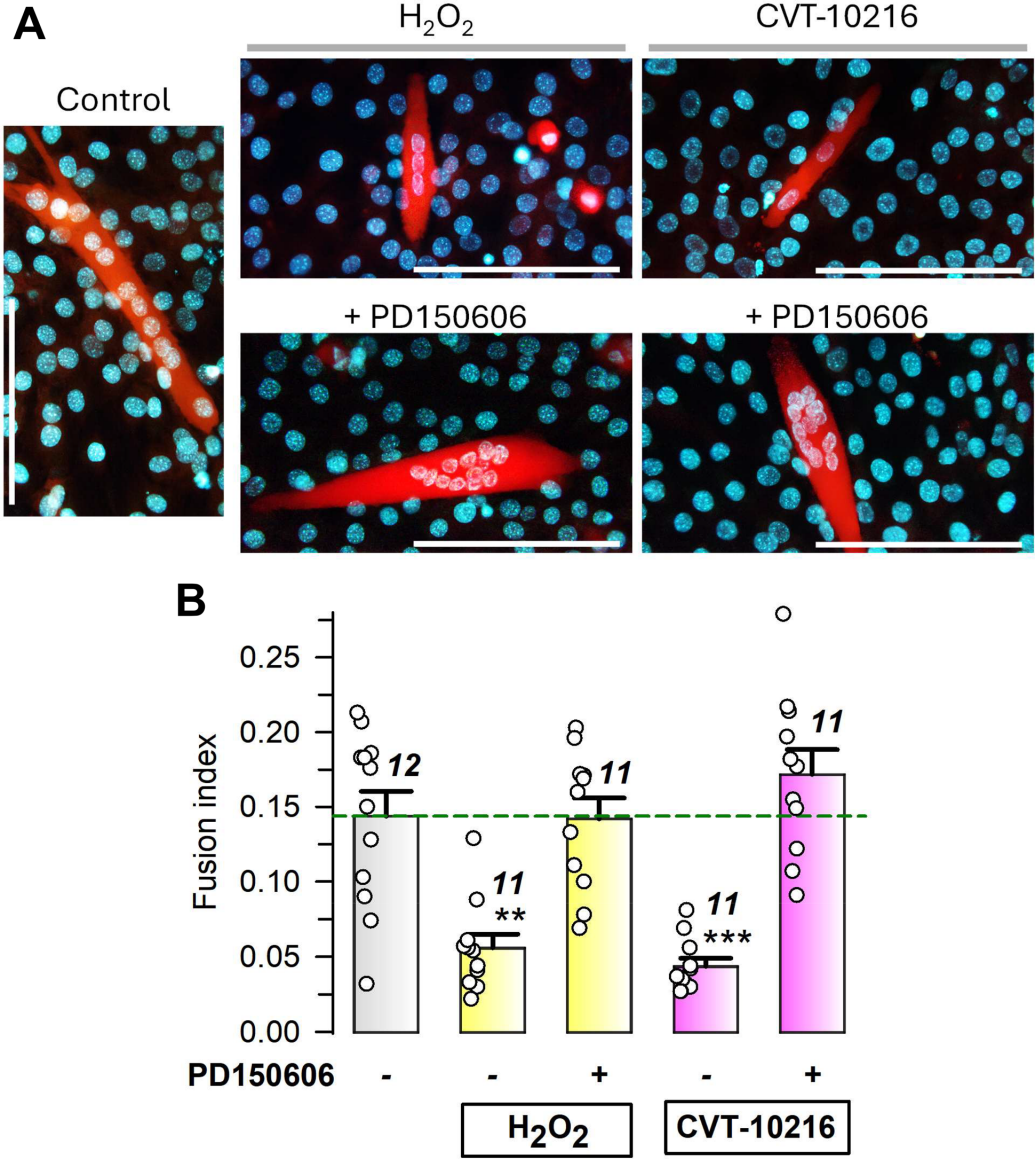
The calpain inhibitor PD150606 prevents the early down-regulation of myoblast fusion by H_2_O_2_ and CVT-10216. **A**) Representative images of myotubes cultured for three days under control conditions, with H_2_O_2_ (1 µM), with CVT-10216 (1 µM), or with each compound in combination with PD150606 (30 µM). The calibration bars stands for 150 µm. **B**) Fusion index values obtained from cells cultured as described in **A**. ^**^p < 0.005, ^***^p < 0.001, compared to control

Previous research suggests that calpain activation mediates early H_2_O_2_-induced inhibition of myotube formation (24 h of differentiation [19]). Thus, we set out to investigate whether a similar mechanism contributes to ALDH2 effects. As shown in Fig. 9, H_2_O_2_ significantly reduced the fusion index (by 60%, see also Fig. 6). Likewise, the ALDH2 antagonist CVT-10216 also reduced the fusion index, in this case by 70% (Fig. 9, *CVT-10216;* like daidzin, see Fig. 7). Nevertheless, coincubation with the calpain inhibitor PD150606 completely prevented these inhibitory effects (Fig. 9). Thus, both ALDH2 inhibition and ROS overproduction apparently recruit calpain, and the latter appears to be responsible for the corresponding myotube atrophy during early myogenesis.

### Relevance of ALDH2 and oxidative stress for the late stage of myogenesis

**Fig. 10:**
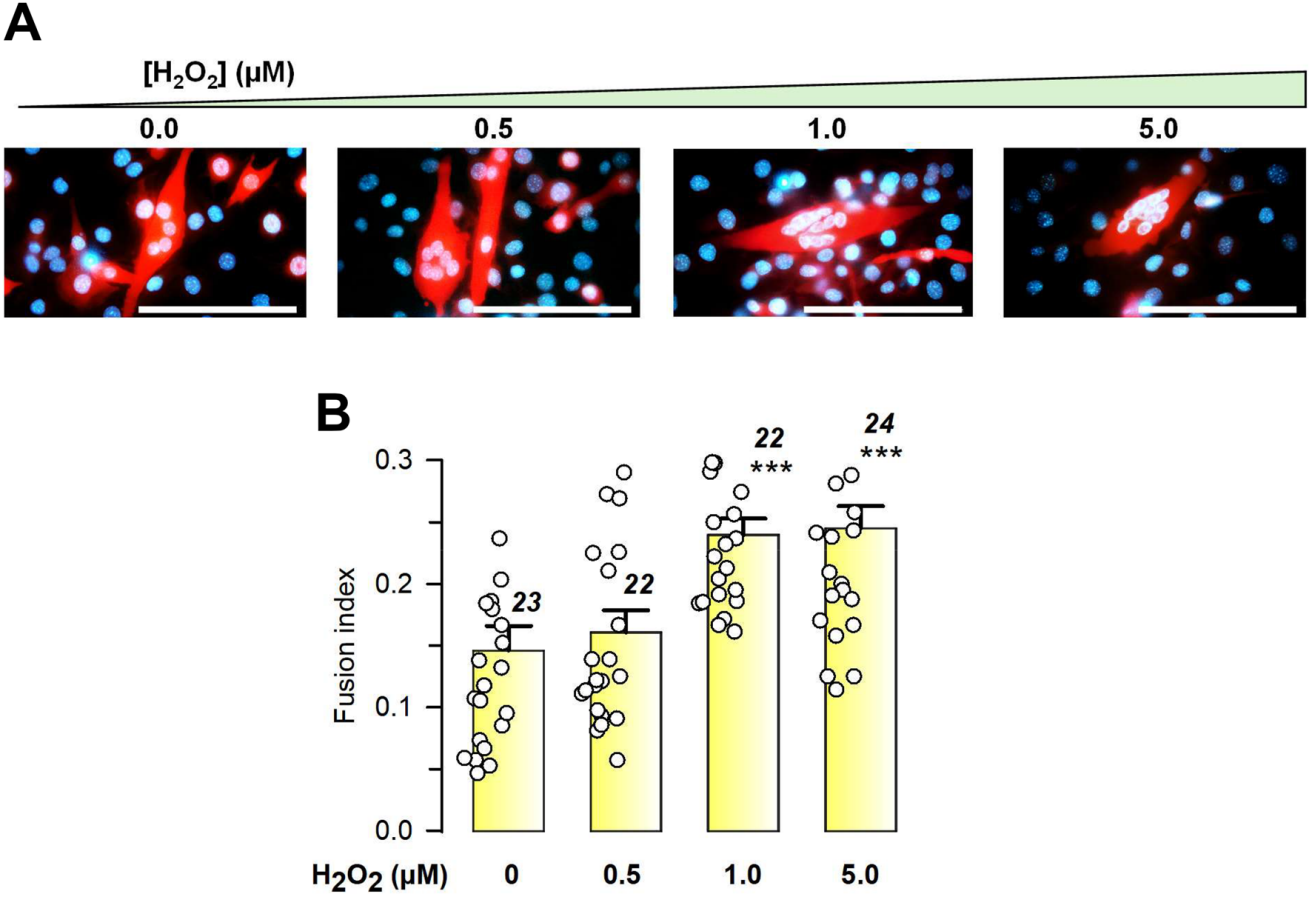
Late stimulation of myotube formation by oxidative stress. **A**) Representative photos of calcein and Hoechst fluorescence that were obtained from cells exposed for 4–5 days (i.e., from d4 to d8-d9) to different concentrations of H_2_O_2_. Calibration bars stand for 150 µm. **B**) Average values of fusion index estimated from experimental conditions described in **A**. ^***^P < 0.001, compared to 0 µM

The results of Fig. 5 indicate that the redox state can be regulated by days 4–5 of the late differentiation stage. We therefore decided to assess the possible and consequent long-term effects on myotube formation (i.e., by days 8–9). Results derived from incubating cells with hydrogen peroxide are shown in Fig. 10. Remarkably, in contrast to the early H_2_O_2_ inhibitory effect on myogenesis (Fig. 6), exposing cells to H_2_O_2_ (1–5 µM) from d4 to d8-9 promoted a 1.65–fold increase in fusion index (Fig. 10). This late stimulatory action is in accord with a recent report by our group showing that oxidative stress enhances myoblast fusion in myotubes of 8–9 days, through a signaling pathway that relies on IL-6 overexpression and corresponding autocrine mechanisms [29].

**Fig. 11:**
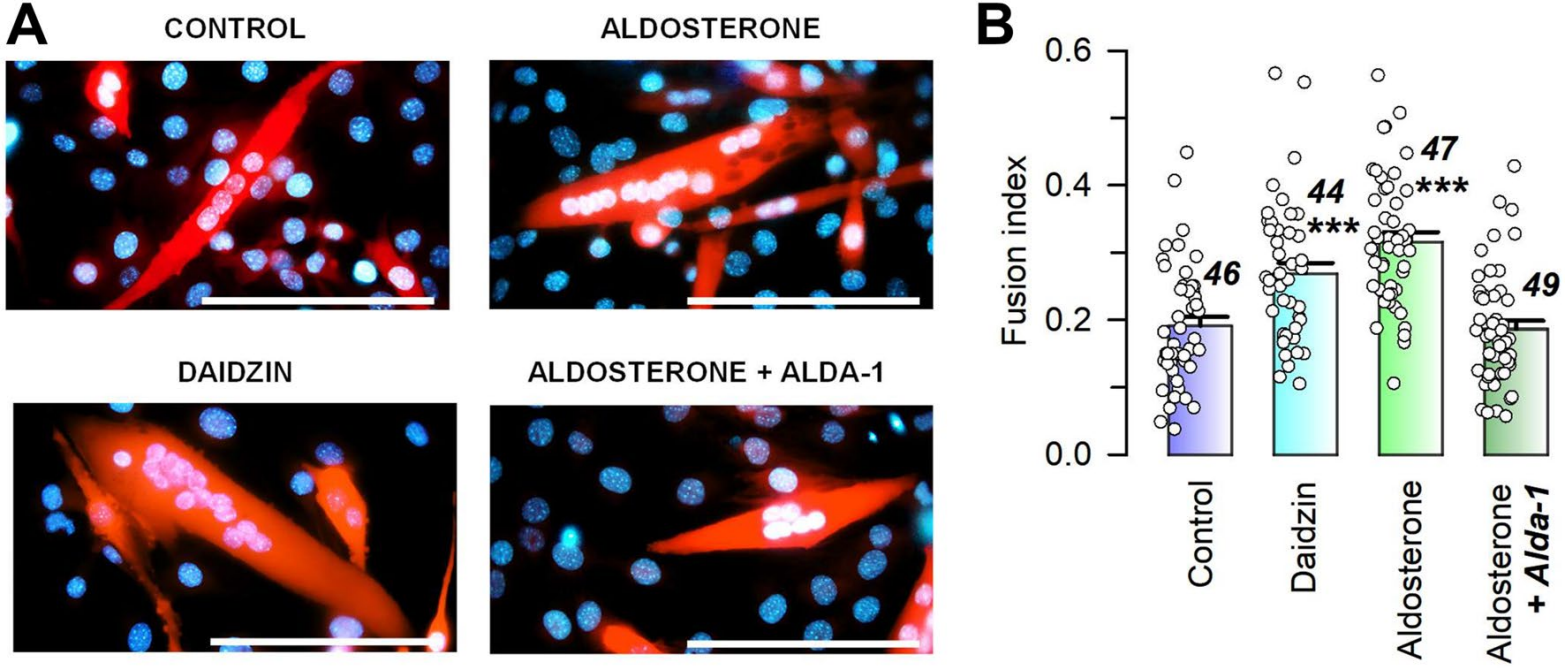
Aldosterone and daidzin replicate the H_2_O_2_-induced stimulation of myoblast fusion during the late stage of myogenesis, while Alda-1 inhibits this response. **A**) Images of cells cultured as in Fig. 10A, but under either control conditions or the presence of daidzin (20 µM), and aldosterone (100 nM). A subgroup of aldosterone-treated cells was also exposed to Alda-1 (20 μM). Calibration bars are equivalent to 150 µm. **B**) Average values of fusion index for cells cultured as in **A**. ^***^p < 0.001, compared to control

As we have been mentioning, it is well known that aldosterone induces pro-oxidant conditions (e.g., Fig. 4; [17, 22]) and thereby reproduces the inhibition of myogenesis by pro-oxidant molecules, in the first 3 days of differentiation (Figs. 7 and 8). In the next experiments, we studied if this hormone could also mimic the H_2_O_2_ stimulatory effect on the late model of differentiation (as shown in Fig. 10). The corresponding results indicate that this is the case, as the hormone increased the fusion index to a similar extent as H_2_O_2_ (i.e., up to ~ 60%; Fig. 11, *Aldosterone*).

We next studied the possibility that Alda-1, which is able to both prevent ROS overproduction (see Figs. 2, 3, 4 and 5) and enhance myoblast fusion in aldosterone-treated cells during the first 3 days of differentiation (see above), could also prevent the late and positive effect of aldosterone on myotube formation. Remarkably, Alda-1 indeed prevented aldosterone from stimulating the advanced phase of myotube formation (Fig. 11, *Aldosterone* + *Alda-1*). This data provides further support for the view that ALDH2 activity is critical for skeletal muscle development. Additional evidence was obtained with daidzin. If ALDH2 were constitutively active throughout the late processes of myogenesis, then exposing cells to daidzin from d4 to d8-9 should stimulate myotube formation (like H_2_O_2_ and aldosterone; Figs. 10 and 11, respectively). In keeping with this view, daidzin promoted a 40% increase in the fusion index compared to controls (Fig. 11 – *Daidzin*).

**Fig. 12:**
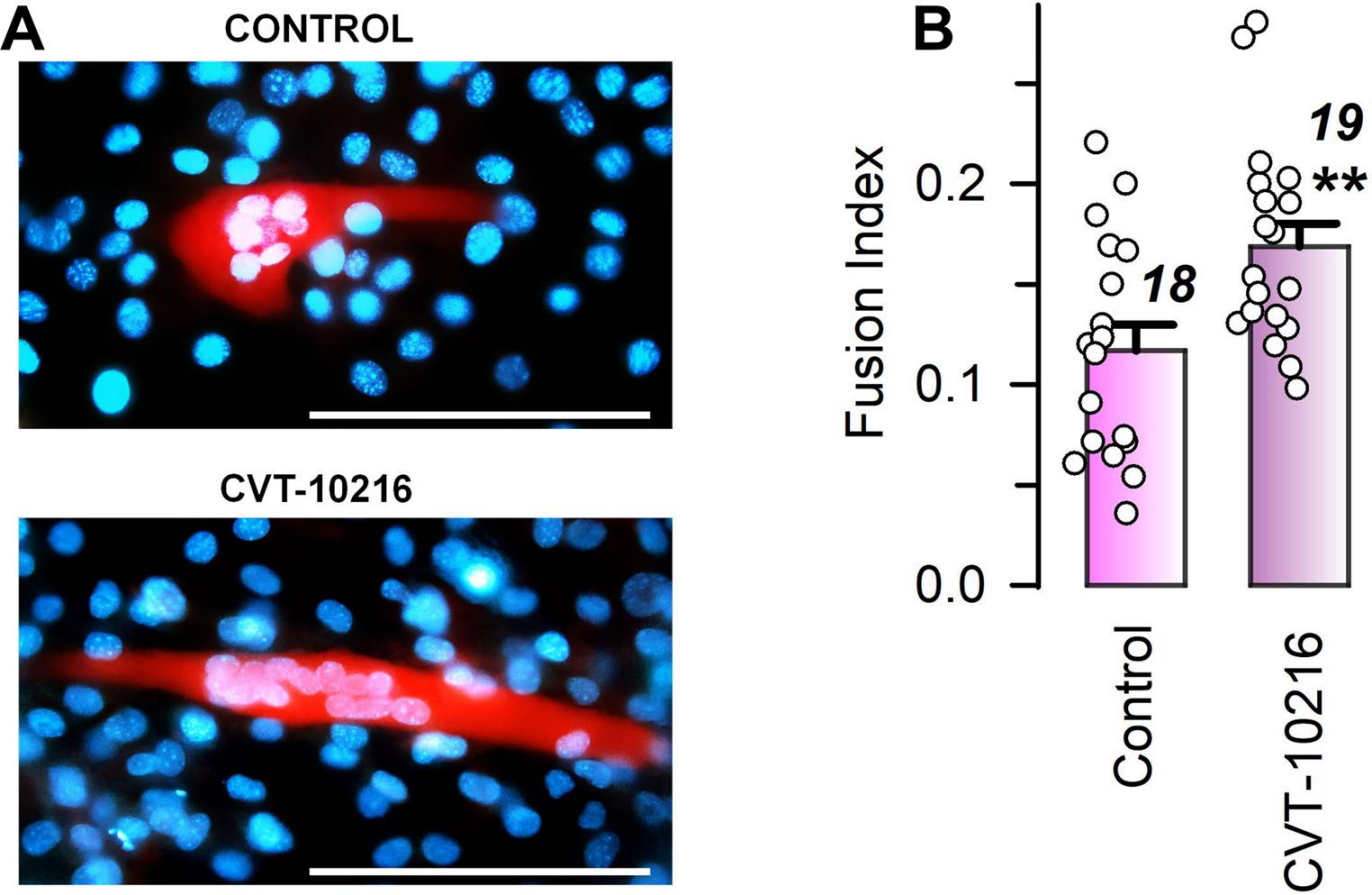
The ALDH2 antagonist CVT-10216 enhances myotube formation in the late stage of differentiation. **A**) Examples of images obtained from cells cultured 8-9d under either control conditions or the presence of 1 µM CVT-10216 (from d4). The scale bars stand for 150 µm. **B**) Fusion index obtained from myotubes cultured as in **A**. ^**^P < 0.01

In Fig. 12, we investigated whether the stimulation of myoblast fusion by H_2_O_2_, aldosterone, and daidzin in the late model of myogenesis (shown in Figs. 10 and 11) could be reproduced by the ALDH2 antagonist CVT-10216 (which inhibited myoblast fusion in the early model, see Fig. 9 – *CVT-10216*). Notably, cells treated with 1 µM of this compound also exhibited a marked increase in fusion index (1.45-fold, Fig. 12).

Therefore, both ALDH2 inhibitors promote a late increase in myoblast fusion of approximately 40–45% (Fig. 11–*Daidzin* and Fig. 12–*CVT-10216*), probably by substrate accumulation (4-HNE) and subsequent ROS overproduction (Figs. 2, 3, 4 and 5). ALDH2 thus appears to be catalytically active and to influence myoblast fusion at both early and late stages of myogenesis.

**Fig. 13:**
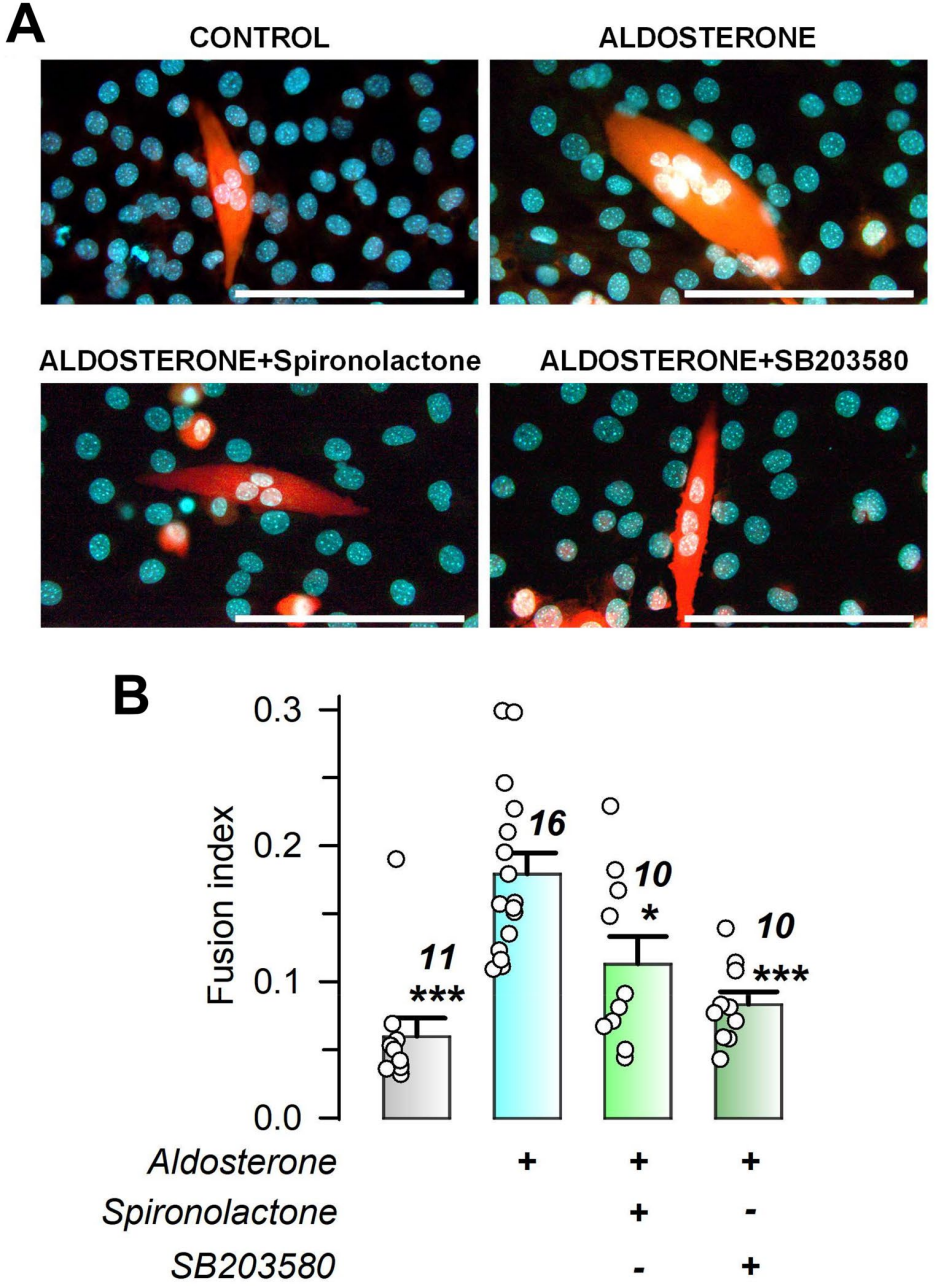
The late stimulation of myotube formation by aldosterone requires activation of MR and p38-MAPK. **A**) Representative microphotographs of cells cultured from d4 to d8 in control conditions or the presences of 100 nM aldosterone (*top*). Subgroups of the latter (*bottom*) were also incubated with either spironolactone (1 µM) or SB203580 (10 µM). Calibration bars, 150 µm. **B**) Fusion index values obtained for cell groups as those shown in **A**. ^*^p < 0.05, ^***^p < 0.001, compared to aldosterone

Our next goal was to find other factors involved in the late stimulation of myotube formation. We previously showed an autocrine mechanism involving the IL-6/p38/IL-6 signaling, which is responsible for myonuclei accretion during late differentiation [29]. Interestingly, as shown in Fig. 13, aldosterone also activates this process, as the p38-MAPK inhibitor SB203580 blocked the hormone’s effect (Fig. 13, *Aldosterone* + *SB203580*). The same was observed with spironolactone (Fig. 13, *Aldosterone* + *spironolactone*), suggesting that the MR is also involved (as previously shown for the early inhibition of myogenesis [17]).

## Discussion

This study was designed based on previous fundamental studies: the well-known antioxidant effects of ALDH2 [1, 8]; indirect evidence that C2C12 cells express this enzyme [5, 18]; and the demonstration that myoblast fusion can be inhibited and stimulated by ROS, depending on the specific differentiation period (early and late, respectively [17, 29]. All this led us to speculate that if ALDH2 is correctly assembled and enzymatically active, then its pharmacological modulation might regulate both ROS production and myotube formation. To investigate this hypothesis, we: ***1)*** established the two models of early and late differentiation, ***2)*** corroborated that ROS indeed exerts a biphasic regulation on myotube formation, and ***3)*** demonstrated that ROS production and myotube formation can be modulated by aldosterone and four distinct ALDH2 regulatory compounds. Consequently, this work represents the first evidence that ALDH2 regulates ROS production in myogenic cells and, thereby, myotube formation.

Below, we discuss the potential relevance of our findings to physiological and pathological conditions as well as the possible molecular mechanisms involved.

### Molecular mechanisms

The presence of ALDH2 in myogenic cells was suspected since 2011 [5]. Further indirect evidence was also published more than a decade later [18]. In both cases, the conclusion was drawn from proteomic studies, using mass spectrometry analysis. The first study focused on comparing the profile of mitochondrial proteins between proliferating myoblasts and differentiated myotubes. Using two-dimensional electrophoresis and silver staining, the authors identified a spot whose intensity was higher when obtained from myotubes than from myoblasts. After mass spectrometry characterization, they suggested the presence of a putative ALDH2, which was differentially expressed during myogenesis (as part of augmented mitochondrial mass and functionality [5]). In the second one [18], the authors studied protein expression patterns, in the context of myotube atrophy induced by a conditioned medium (CM) derived from colon cancer cells (C26). Using proteomic analysis, they found that, in addition to inhibiting myoblast fusion, the C26-derived CM also alters the expression of many proteins involved in distinct cellular functions (e.g., inflammation, mitochondrial function, and ROS production). In particular, the ALDH2 abundance was inferred to be reduced. However, this interpretation was not corroborated by western blotting, in contrast to the validation performed for other proteomics data [18].

Chronic aldosterone treatment exerts pro-oxidant effects in many cell types, including mesangial [21], adult rat cardiac [22], epithelial [26], and C2C12 (this study, [17]). In the latter, Lee et al. (2021) demonstrated that ROS overproduction and early myotube atrophy both depend on the recruitment of the MR and our present results also suggest that this receptor is involved in the late differentiation stage (Fig. 13). However, the precise mechanism linking MR activation to ROS overproduction remains to be elucidated. An interesting possibility is that activation of the aldosterone-MR system recruits higher NADPH oxidase (NOS) activity through modifying the expression of this enzyme’s subunits. Another possibility is that MR activation may inhibit the expression of proteins involved in antioxidant defense. Experimental support for both mechanisms has been reported in mesangial and epithelial cells [21, 26].

Of note, in our experiments, concentrations of H_2_O_2_ above 5 µM resulted in significant reductions in the total number of nuclei (data not shown), suggesting lethal toxicity. Particularly, treating cells with 25 µM and 50 µM (from d0 to d3) reduced the total number of nuclei by 30% and 60%, respectively, which is why we focused on characterizing only the effects of 0.5–5 µM. A relatively high H_2_O_2_ concentration (300 µM) induces mitochondrial dysfunction and fragmentation without significantly affecting cell viability within 3–5 h [11]. However, in the long term (48–72 h), high H_2_O_2_ concentrations (100–4000 µM) can actually lead to apoptotic cell death [28] and thereby reducing cell viability [19]. Consequently, results on myoblast fusion under extreme oxidant conditions should be interpreted with caution, as they may be influenced by lethal toxicity and corresponding disruptions in cell density and cell-to-cell contact.

Thus far, the mechanisms underlying the regulation of myoblast fusion by ROS remain relatively poorly understood. In the early stage of myogenesis, myotube atrophy resulting from oxidative stress can be prevented by the protease inhibitor leupeptin or by downregulating calpain-1 expression, which led McClung et al. 2009 to propose that calpain-1 plays a critical role in this process [19]. Our data of Fig. 9, showing that a calpain inhibitor (PD150606) prevents the effects of both H_2_O_2_ and CVT-10216 reinforce this notion. McClung et al. 2009 also speculated that oxidative damage to the plasma membrane Ca^2+^-ATPase (previously shown in [27]) might lead to high intracellular Ca^2+^ levels and Ca^2+^-dependent calpain-1 activation [19]. Further work is needed to systematically characterize the role of these molecules.

In our study, a pivotal assumption was made for establishing the two experimental models of early and late differentiation. Specifically, we hypothesized that oxidative conditions should stimulate myoblast fusion exclusively during the late stage of myotube formation. This assumption was based on the following previous observations: ***1)*** Chronic exposure to either H_2_O_2_ or a ROS-producing reagent (pyrogallol) induces a tenfold increase in IL-6 production in differentiated myotubes but not in proliferating myoblasts [15]. ***2)*** The IL-6 promotes an increase in expression levels of muscle-specific differentiation markers (myogenin, α-actin, and creatine kinase [4]. ***3)*** Moreover, we have reported that a p38/IL-6/p38 loop enhances myoblast fusion in cells differentiated by 8–9 days [29]. All this, combined with our present data (see Fig. 13 - *SB203580*) provides compelling evidence that a ROS-dependent IL-6 autocrine system is involved during late processes of myotube formation.

Interestingly, recent evidence suggests that the compound AD-5591 acts more potently than Alda-1 on ALDH2. Specifically, results from in vitro assays indicate that AD-5591 stimulates ALDH2 activity twofold more efficiently than Alda-1 [7]. Initially, this seems inconsistent with our finding that both compounds reduce oxidant stress with similar potency (Fig. 5; IC_50_ values about 260–390 nM). This discrepancy likely arises from methodological differences. For instance, Chang et al. (2023) evaluated the catalytic activity of recombinant human ALDH2 purified from *Escherichia coli* at a single, high concentration of 100 µM, which was 100 times greater than the 1 µM required to inhibit approximately 80% of the aldosterone pro-oxidant effect (Fig. 5). These findings indirectly support a model in which activation of only a small fraction of ALDH2 proteins may be sufficient to disrupt ROS-induced ROS generation. Future research utilizing more compatible methodologies may help resolve this paradox.

Another in vitro study indicates that Alda-1 also alters the activity of c-Jun N-terminal kinase isoform 2 (JNK2). More precisely, Yan et al. 2024 observed that a recombinant JNK2 exhibits 20% lower activity in presence of Alda-1 (20 µM [30]). Even though this appears to be a minor inhibition, the potential contribution of JNK2 to our current findings warrants careful investigation, particularly since it has been reported that pharmacological JNK2 blockade leads to apoptosis and reduced expression/activity of differentiation markers (myosin, myogenin, and creatine kinase [13].

### Possible pathophysiological relevance

The in vivo significance of the biphasic regulation of myoblast fusion by ALDH2 through ROS may depend on physiological contexts, such as metabolic state, disease condition, physical activity, and developmental stage. In this regard, it is interesting that adult ALDH2 activity-deficient transgenic mice develop atrophy, condition that can be prevented with an antioxidant-rich diet [12, 14]. Combined with our present results, this suggests that low ALDH2 activity during early myogenesis limits muscle growth throughout embryogenesis and persists into adulthood.

Remarkably, and as mentioned above, proteomics data suggests that culture medium collected from cancer cells induces myotube atrophy, and this effect is presumably associated with reduced ALDH2 expression [18]. Taken together, these previous findings suggest that the atrophy induced by cancer cells might depend on promoting an oxidative environment, at least partially by inhibiting ALDH2 expression. In reciprocity, and based also on our present results, it seems reasonable to propose that stimulating skeletal muscle ALDH2 should prevent cancer cells from promoting atrophy. Likewise, the present finding that ALDH2 agonists blunt the early aldosterone-induced atrophy suggests that these compounds may be beneficial in certain cases of cachexia linked to hyperaldosteronism [6, 16].

Our findings on the late phase of differentiation could be also relevant for pathological conditions. Particularly, because the p38– and IL–6–dependent upregulation of myoblast fusion is thought to explain an altered myonuclei distribution in hereditary muscle diseases [29] and thereby contribute to understanding the corresponding muscle weakness as the nuclei’s subcellular location is critical to proper sarcomere organization [2].

On the other hand, IL-6 is considered highly significant for physiological conditions, due to its hypertrophic effects in response to muscle exercise [23, 25]. The following evidence indirectly suggest that the ROS-induced IL-6 release and the ensuing improvement in myotube formation could also be relevant in this context. We have been mentioning that oxidative stress enhances the secretion of this myokine, selectively in myotubes (as opposed to myoblasts; [15]). It is also important to note that both mouse primary and C2C12 myotubes elevate ROS production in response to electrical stimulation. Particularly, 12.5 s and 60 min of stimulation enhance ROS production by 2.5- and tenfold respectively [9, 24]. Moreover, the electrical stimuli also elevate levels of IL-6 mRNA and secreted protein, which can be prevented by both Ca^2+^-chelating agents and NADPH oxidase inhibitors [9].

**Fig. 14:**
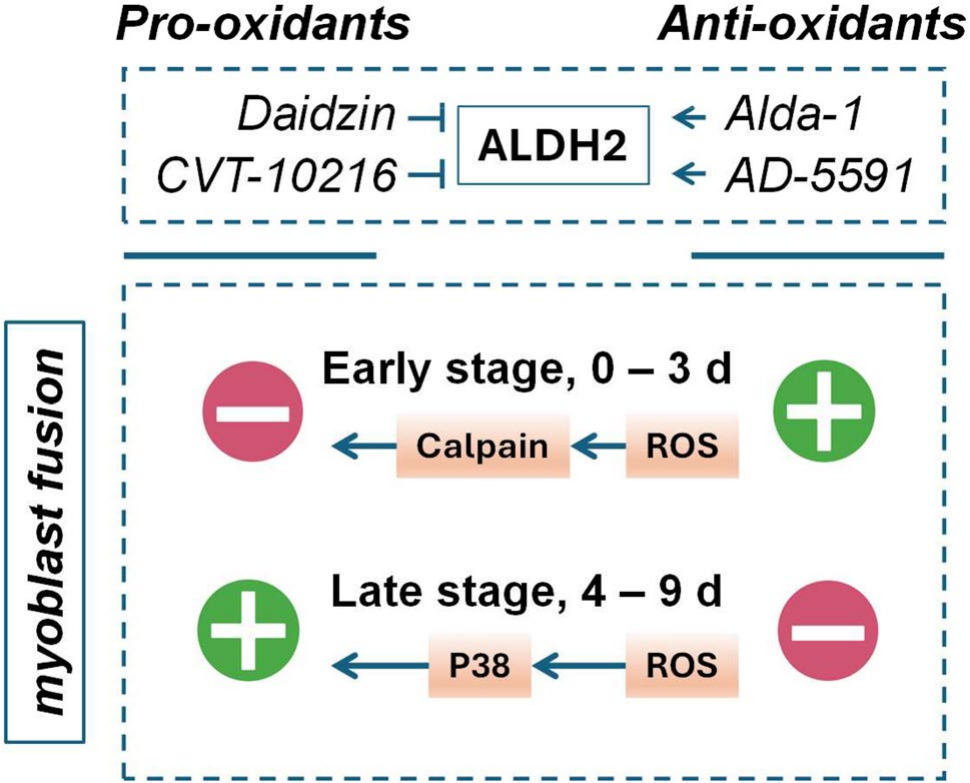
Model of two-phase regulation of myoblast fusion. Classical *Pro-oxidants* (including H_2_O_2_, aldosterone, and 4-HNE) as well as ALDH2 antagonists (Daidzin and CVT-10216) inhibit and stimulate the *Early* and *Late* phases of myotube formation respectively, by upregulating ROS production. Remarkably, ALDH2 agonists (Alda-1 and AD-5591) readily counteract ROS overproduction by these classical *Pro-oxidants* and thereby block the corresponding biphasic modulation of myogenesis. Downstream of ROS, *Calpain* and *p38-MAPK* mediate early inhibition and late stimulation, respectively. Although not shown, *Calpain* is also involved in late processes [29]

Regardless of its relevance to in vivo conditions, this study reveals an important role for ALDH2 in myogenic cells and reconciles apparently contradictory observations by characterizing the biphasic effects of redox state during myogenesis. Therefore, in Fig. 14 we summarize our main findings, framed in the context of the two phases of myotube differentiation (early- and late-stage; Fig. 14).

## Conclusion

In summary, ALDH2 can be immunodetected in myogenic cells, and its pharmacological modulation is critical for shaping both oxidative state and myoblast fusion. ROS signaling pathways promote a bidirectional regulation of myoblast fusion, consisting of early inhibition (d0-d3) and late stimulation (d4-d9). Although the precise mechanisms linking ROS to the regulation of myoblast fusion have yet to be firmly established, our data combined with others point to calpain-1 participation during the first three days of differentiation, while a p38/IL-6/p38 autocrine system is most likely involved in later stages. Further research is required to define the specific roles of these and other intermediaries.

## Supplementary Information

Below is the link to the electronic supplementary material.

Supplementary file1 (PDF 333 KB)

## Data Availability

All data supporting the findings of this study are available within the paper.
