# Supplementary material for: Biphasic regulation of myogenesis by ALDH2, aldosterone, and oxidative stress
Source: Pflugers Arch. 2026 Jun 9;478(6):56. doi: 10.1007/s00424-026-03185-w (PMC13246838; doi:10.1007/s00424-026-03185-w)
Supplement: Supplementary file 1 — Supplementary file1 (PDF 333 KB) [file 424_2026_3185_MOESM1_ESM.pdf]

SUPPLEMENTARY MATERIAL

Biphasic regulation of myogenesis by ALDH2, aldosterone, and oxidative stress.

José Emmanuel Martínez-Cortés[1], Maikel Valle-Clara[1],

Damaso Fernández-Hernández[1],

and Guillermo Ávila[1] *

[1] Departamento de Bioquímica, Cinvestav.

México City, México

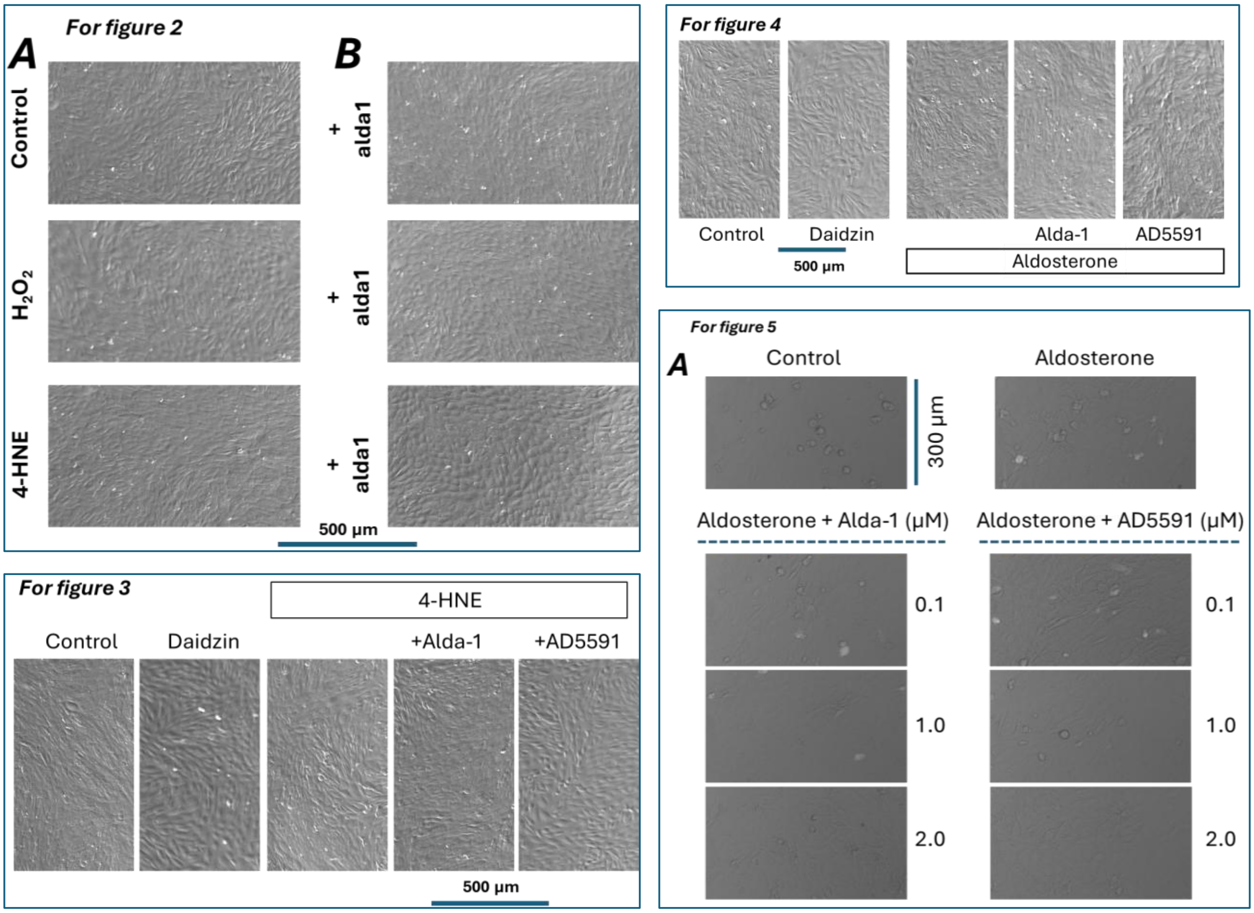

Figure. S1. Images acquired using relief-contrast optics for the same cell cultures as in Figures 2-5, either with 4x (for Figs. 2–4) or 20x objectives (for Fig. 5).

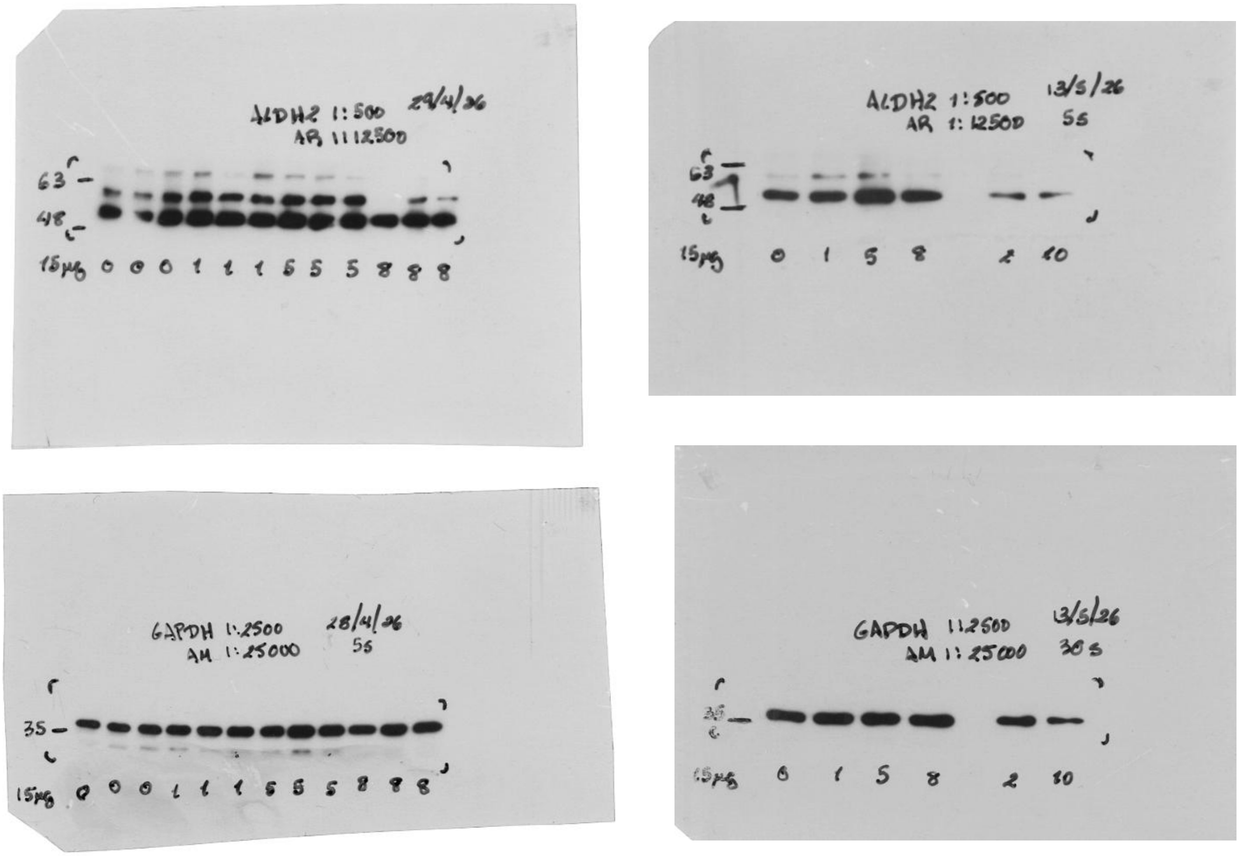

Figure. S2. Uncropped blots used for densitometric analysis of Fig 1C.
